# Modulation of Pork Fatty Acid Composition Through the Inclusion of Stearic Acid and Palm Oil in Growing–Finishing Pigs Diet

**DOI:** 10.1002/fsn3.71342

**Published:** 2026-01-05

**Authors:** Vetriselvi Sampath, Eunju Ko, Jong Sang Yoo, Jemin Ahn, In Ho Kim

**Affiliations:** ^1^ Department of Animal Biotechnology Dankook University Cheonan South Korea; ^2^ Smart Animal Bio Institute Dankook University Cheonan South Korea; ^3^ Department of Research & Development of Central Institute Daehan Feed Company Incheon Republic of Korea

**Keywords:** belly lean, loin lean, pork belly, saturated fatty acid

## Abstract

This study aims to evaluate the effect of stearic acid (SA) and palm oil (PO) on growth performance, carcass characteristics, and fatty acid composition in pork of growing‐finishing pigs. A total of 150 pigs were assigned to three dietary treatments for 12 weeks: control (CON; basal diet), SA (CON + 0.50% SA), and PO (CON + 2% PO), with 10 replicates of 5 pigs/pen. Growth performance, backfat thickness, carcass weight, and meat quality were not significantly influenced by dietary treatments (*p* > 0.05). However, fatty acid (FA) analysis revealed that pigs fed the SA diet led to a consistent increase in SA (C18:0) content across pork belly fat, belly lean, and loin lean tissues (*p* < 0.05), resulting in significantly higher total saturated fatty acid levels and lower total unsaturated fatty acid concentrations. In contrast, the PO group showed increased monounsaturated fatty acids (MUFA), particularly oleic acid (C18:1c), and improved the MUFA/SFA ratio (*p* < 0.05) in all samples. Although polyunsaturated fatty acid (PUFA) content and the ω‐6:ω‐3 ratio were not significantly affected by treatments (*p* > 0.05), the PUFA/SFA ratio tended to be higher in the PO group, especially in pork loin lean (*p* = 0.085). The iodine value was significantly reduced in the SA group across all tissue types, confirming a more saturated lipid profile (*p* < 0.05). In summary, the SA and PO treated groups had no impact on growth performance or meat quality traits, but they markedly altered the fatty acid composition of pork. SA promoted a firmer, more saturated fat profile, potentially beneficial for processing traits but less favorable nutritionally. Palm oil (PO), on the other hand, supported a more unsaturated fat profile with improved nutritional attributes. These findings provide practical insights into swine diet formulation aimed at optimizing pork quality based on specific processing or health‐oriented goals.

## Graphical abstract

Modulation of pork fatty acid composition through the inclusion of stearic acid (SA) and palm oil (PO).

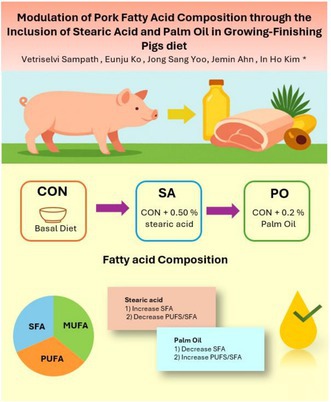

## 1 Introduction

Pork meat is a valuable source of essential nutrients and energy, and its quality has significant implications for human health. It provides a wide array of nutrients, including high‐quality proteins, lipids, fatty acids, minerals, and vitamins (Pereira and Vicente 2013). The proteins in pork are composed of amino acids, including essential amino acids that cannot be synthesized by the human body and must therefore be obtained through diet (Wu et al. 2014). In recent years, the manipulation of fatty acid (FA) composition in muscle and adipose tissue has garnered considerable interest as a strategy to improve the nutritional qualities of meat (Wood et al. 2004; Gutiérrez‐Luna et al. 2022). In addition to health implications, FA composition influences key meat quality parameters such as tissue firmness, oxidative stability, shelf life, and sensory attributes, particularly flavor (Isabel et al. 2003; Mazzoleni et al. 2023). Indeed, the quality of pork meat is highly influenced by multiple factors, including genetics, management practices, and notably, dietary composition, particularly the source and type of dietary fat.

Dietary lipids not only enhance energy density in swine diets but also play a crucial role in regulating carcass fat deposition, muscle composition, and overall meat quality attributes such as pH, color, and intramuscular fat content (Marco Tretola et al. 2024). Among dietary fats, PO and SA present distinct fatty acid profiles and potential benefits, and some research has yielded mixed findings regarding their effects on pork fatty acid composition. For instance, PO, widely used in animal feed, is rich in saturated and monounsaturated fatty acids, especially palmitic acid, which enhances dietary energy and promotes lipid deposition in tissues (Bahurmiz and Ng 2007; Theophillus Olayiwola et al. 2011). On the other hand, SA, a long‐chain saturated fatty acid, is less digestible and is associated with minimal effects on plasma cholesterol and fat accumulation due to its lower intestinal absorption (Bonanome and Grundy 1988). These differences suggest that the metabolic processing and tissue deposition of fatty acids from SA and PO may vary, thereby potentially affecting pork quality traits. Based on earlier literature, we hypothesize that pigs fed diets containing SA are expected to produce leaner carcasses with firmer fat, whereas PO addition may lead to changes in enhanced fatty acid absorption. Also, to our knowledge, there have been very limited studies on using these two lipid sources in pigs, and thus we intend to examine the effect of SA and/or PO in the diet of growing‐finishing pigs on growth performance, carcass traits, meat quality, and the fatty acid profile of pork.

## 2 Materials and Methods

### 2.1 Ethics

This research was complied with the ARRIVE guidelines and carried out in accordance with the Institutional Animal Care and Use Committee (IACUC) of Dankook University (DKU), South Korea. The experimental protocol (DK‐1‐2420), detailing the management and care of animals, was reviewed and approved by the IACUC of Dankook University, prior to the start of the experiment.

### 2.2 Animals, Diets, and Husbandry Management

A total of 150 hybrid ([Yorkshire × Landrace] × Duroc) growing‐finishing pigs with the initial body weight of 37.46 ± 2.31 were used in a 12 week trial. All animals were divided into three groups with 10 replicates of 5 pigs per pen and were fed with one of three diets. The experimental diets include control (CON), basal diet; stearic acid (SA); CON + 0.50% SA; and palm oil (PO), CON + 2% PO. The composition and proximate analysis of the diets are shown in Table 1. Basal diet was formulated according to NRC (2012) regulation. The SA and PA were commercially obtained from Daehan Feed Co. Ltd. (Incheon, Republic of Korea). The barn was fully enclosed, climate‐controlled with mechanical ventilation. The pens had adjustable gates facing the alleyway and allowed 0.93 m^2^/pig. Each pen was equipped with a waterer and dry self‐feeder with two eating spaces located in the fence line. Each feeder hole was 35.6 cm in length with a 27.9 cm horizontal depth. Pens were located over a completely slated concrete floor with a 1.20 m pit underneath for manure storage. The room temperature was fixed at 24°C. Feeder positions were monitored daily and all animals had *ad libitum* access to water and a mash form of feed.

**TABLE 1:** Basal diet composition (as fed basis).

Ingredients (%)	Growing			Finishing		
	Basal diet	Stearic acid	Palm oil	Basal diet	Stearic acid	Palm oil
Corn	59.20	53.27	59.20	69.00	63.67	69.00
Soybean meal	18.53	15.47	18.53	11.30	9.00	11.30
Rapeseed meal	2.67	6.67	2.67	5.00	7.00	5.00
Distrillers dried grain solubles	6.67	6.67	6.67	5.00	6.00	5.00
Palm kernel meal	2.67	4.00	2.67	2.00	3.50	2.00
Animal fat	4.00	4.80	2.00	4.00	4.70	2.00
Palm oil	—	—	2.00	—	—	2.00
Molasses	2.00	3.33	2.00	0.50	2.00	0.50
Limestone	1.47	2.00	1.47	1.10	1.20	1.10
Mono di‐calcium phosphate	0.40	0.91	0.40	0.47	0.73	0.47
Salt	0.67	0.67	0.67	0.50	0.50	0.50
Methionine (98%)	0.15	0.13	0.15	0.03	0.03	0.03
Lysine (55%)	0.77	0.77	0.77	0.54	0.59	0.54
Threonine (98%)	0.15	0.16	0.15	0.08	0.09	0.08
Tryptophan (98%)	0.37	0.37	0.37	0.23	0.24	0.23
Choline (25%)	0.05	0.05	0.05	0.05	0.05	0.05
Copper sulfate	0.03	0.03	0.03	—	—	—
Stearic acid	—	0.50	—	—	0.50	—
Min/Vit mix^1^	0.20	0.20	0.20	0.20	0.20	0.20
Total	100.00	100.00	100.00	100.00	100.00	100.00
Calculated value						
Moisture, %	12.26	12.93	12.26	12.38	12.87	12.38
Crude Protein, %	15.86	16.35	15.86	14.40	14.40	14.40
Crude Fat, %	6.47	7.48	6.47	6.74	7.86	6.74
Crude Fiber, %	3.28	3.46	3.28	3.14	3.31	3.14
Crude Ash, %	5.08	6.04	5.08	4.48	6.04	4.48
Net Energy, kcal/kg	2505	2489	2505	2518	2505	2518
Saturated fatty acid	18	21	19	19	24	20
Iodine value	116	110	115	117	110	116
Lysine, %	1.08	1.08	1.08	0.89	0.89	0.89

^1^ Provided per kg diet: Fe, 100 mg as ferrous sulfate; Cu, 17 mg as copper sulfate; Mn, 17 mg as manganese oxide; I, 0.5 mg as potassium iodide; and Se, 0.3 mg as sodium selenite; vitamin A, 10,800 IU; vitamin D3, 4000 IU; vitamin E, 40 IU; vitamin K3, 4 mg; vitamin B1, 6 mg; vitamin B2, 12 mg; vitamin B6, 6 mg; vitamin B12, 0.05 mg; biotin, 0.2 mg; folic acid, 2 mg; niacin, 50 mg; D‐calcium pantothenate, 25 mg.

### 2.3 Slaughter and Sampling Procedures

Throughout the experimental period, individual body weights of pigs were recorded, while feed intake and disappearance were monitored on a pen basis to calculate average daily gain (ADG), average daily feed intake (ADFI), and feed conversion ratio (FCR). At the end of week 12, backfat thickness (BFT) was assessed using a Piglog 105 ultrasound device (Denmark). Following this, 10 pigs per treatment group were randomly selected, transported to a commercial slaughter facility, and held for 6 h under fasting conditions with free access to water. Slaughter was performed following CO_2_ stunning. After slaughter, carcasses were chilled for 20 h at ±4°C, weighed, and transferred to the laboratory. The longissimus thoracis et lumborum (LTL) muscle was collected from the left side of each carcass. Visible fat and connective tissues were removed, and subsamples were prepared for further analyses. Within 24 h post‐mortem, water‐holding capacity (WHC), cooking loss, and drip loss were measured. Approximately 300 g of each muscle sample was stored at −20°C for fatty acid (FA) profiling. WHC was assessed by placing 0.3 g of meat on 120 mm filter paper, pressing it for 3 min, and measuring the area of meat and exuded moisture using a digital area‐line sensor (MT‐10S, M.T. Precision Co. Ltd., Tokyo, Japan). A lower water‐to‐meat area ratio indicated better WHC. Drip and cooking losses were determined using the procedure of Choe et al. (2017). For fatty acid profiling, the fat extraction was carried out using a chloroform: methanol (2:1) mixture. Samples (15 g) were ground, mixed with 150 mL of solvent, and homogenized at 300 × g for 3 min using a PT‐MRC2100 homogenizer (Littau, Switzerland). The mixture was filtered (Whatman filter paper), and 20 g of sodium sulfate was added to remove moisture. The fat layer was then transferred to a titration flask and dried at 55°C using a rotary evaporator. The residue was derivatized with 1 mL tricosylic acid and 1 mL of 0.5 N sodium hydroxide to produce fatty acid methyl esters (FAME). Later, 1.0 mL of the FAME sample was transferred to an auto‐sampler vial and analyzed using a gas chromatography system with a flame ionization detector (GC‐FID, Columbia, MD, USA), equipped with an Omega wax capillary column (30 m length, 0.25 mm internal diameter, 0.25 μm film thickness). The oven temperature was programmed to start at 50°C (held for 1 min), ramp to 200°C at 25°C/min, and then to 230°C at 5°C/min. Injection and detector temperatures were both maintained at 250°C. Individual fatty acids were identified by comparing their retention times to known standards and expressed as percentages of the total FA content. The n‐6/n‐3 polyunsaturated fatty acid (PUFA) ratio was calculated, along with specific FA ratios: C16:1/C16:0, C18:1/C18:0, C20:3 n‐6/C18:2 n‐6, C22:5 n‐3/C22:6 n‐3, and C20:4 n‐6/C18:2 n‐6, as described by Boschetti et al. (2016).

### 2.4 Data Analysis

Experimental data were investigated using SPSS software (version 18.0, IBM Corp., Armonk, NY, USA). Data on growth performance, backfat thickness, meat quality, and fatty acid profile were subjected to a one‐way analysis of variance (ANOVA) to evaluate the effects of diets. Each pen was considered the experimental unit for growth performance parameters, while individual animals were treated as the experimental units for carcasses, meat quality traits, and fatty acid profiling. Turkey's *b* test was employed for post hoc comparisons between treatment means. Data are presented as means with their corresponding standard error of the mean (SEM). A *p*‐value < 0.05 was considered statistically significant.

## 3 Results

The effects of SA and PO on growth performance, backfat thickness, and meat quality traits of growing‐finishing pigs are presented in Table 2. During the 12 week experimental period, there were no notable differences (*p* > 0.05) found among the treatment groups in any of the measured growth parameters, including final body weight, ADG, ADFI, and FCR. Furthermore, BFT and carcass weight were not significantly affected by the dietary treatments. In terms of meat quality parameters, no significant differences were found among the groups in water holding capacity, cooking loss, or drip loss (*p* > 0.05).

**TABLE 2:** Effect of stearic acid vs. palm oil on growth performance, backfat thickness, and meat quality in growing‐finishing pigs.

Items	CON	SA	PO	SEM^2^	*p*
Final body weight, kg	114.35	113.97	113.05	1.63	0.793
Daily gain, g	845	841	831	18	0.843
Daily feed intake, g	2387	2382	2369	31	0.904
Feed Conversion Ratio (pen basis)	2.833	2.839	2.854	0.031	0.898
Backfat thickness, mm	20.44	20.28	20.07	0.10	0.814
Carcass weight (kg)	91.50	91.34	91.10	0.87	0.945
Water holding capacity, %	52.09	52.77	50.81	0.61	0.452
Cooking loss, %	31.88	31.75	32.22	0.50	0.935
Drip loss, %					
d1	4.06	4.00	4.15	0.09	0.843
d3	11.51	11.16	12.02	0.3	0.535
d5	16.70	16.24	17.38	0.31	0.364
d7	21.76	21.18	22.66	0.36	0.262

*Note:* Control (CON), basal diet; Stearic acid (SA); CON+0.50% SA; Palm oil (PO), CON+2% PO. SEM, Standard Mean Error.

The effects of SA and PO on the fatty acid composition of pork belly fat of growing‐finishing pigs are summarized in Table 3. Among individual fatty acids, stearic acid (C18:0) level was significantly higher (*p* = 0.009) in the SA group (12.77%) compared to both the CON (11.11%) and PO (10.62%) groups. Also, eicosanoic acid (C20:0) showed a tendency (*p* = 0.054) to increase in the SA group (0.17%). The content of linoleic acid (C18:2n6c) was numerically lower in the SA group and higher in the PO group; however, this difference was marginally nonsignificant (*p* = 0.107). Furthermore, pigs fed diet containing SA had a significantly higher proportion of total saturated fatty acids (ΣSFA) (*p* = 0.031) and a lower proportion of total unsaturated fatty acids (ΣUSFA) (*p* = 0.030) compared to those fed PO and CON diets. Additionally, the monounsaturated to saturated fatty acid ratio (MUFA/SFA) tended to be lower in the SA group (1.12) than in CON (1.18) and PO (1.20) groups (*p* = 0.065), indicating a shift toward a more saturated fat profile. However, there were no significant effects observed on the most short‐ and medium‐chain fatty acids (e.g., C6:0 to C14:0), nor on PUFA such as eicosapentaenoic acid (EPA, C20:5n3) and docosahexaenoic acid (DHA, C22:6n3) (*p* > 0.05). The PUFA/SFA ratio and ω‐6: ω‐3 PUFA ratio were also not significantly different among treatments (*p* = 0.168 and *p* = 0.153, respectively). But the iodine value, an indicator of unsaturation, was significantly lower in the SA group (59.07) compared to the other two groups, suggesting a trend toward reduced fat unsaturation with the SA diet.

**TABLE 3:** Effect of stearic acid vs. palm oil on fatty acid profile (pork belly, fat) in growing‐finishing pigs.

Items	CON	SA	PO	SEM^2^	*p*
C4:0	0.00	0.00	0.00	—	
C6:0	0.05	0.05	0.07	0.01	0.325
C8:0	0.03	0.03	0.03	0.003	0.856
C10:0	0.06	0.07	0.06	0.003	0.687
C11:0	0.00	0.00	0.00	—	—
C12:0	0.32	0.33	0.31	0.01	0.447
C13:0	0.00	0.00	0.00	—	—
C14:0	1.80	1.83	1.81	0.03	0.750
C14:1	0.03	0.03	0.03	—	—
C15:0	0.11	0.13	0.13	0.03	0.850
C15:1	0.00	0.00	0.00	—	—
C16:0	25.96	25.95	25.93	0.30	0.998
C16:1	2.78	2.59	2.63	0.10	0.626
C17:0	0.38	0.36	0.39	0.03	0.827
C17:1	0.27	0.24	0.23	0.02	0.299
C18:0	11.11^b^	12.77^a^	10.62^b^	0.39	0.009
C18:1, t	0.04	0.03	0.02	0.01	0.814
C18:1, c	42.89	42.58	43.45	0.26	0.123
C18:2n6t	0.01	0.00	0.02	0.01	0.296
C18:2n6c, LA	11.28^ab^	10.22^b^	11.48^a^	0.34	0.107
C18:3n6	0.01	0.01	0.02	0.01	0.438
C18:3n3, ALA	0.51	0.49	0.48	0.05	0.897
C20:0	0.15^b^	0.17^a^	0.16^ab^	0.01	0.054
C20:1	1.01	1.03	0.89	0.07	0.268
C20:2	0.42	0.40	0.43	0.02	0.698
C20:3n6	0.06	0.05	0.05	0.01	0.705
C21:0	0.00	0.00	0.00	—	—
C20c	0.09	0.07	0.08	0.01	0.394
C20:4n6	0.05	0.04	0.04	0.01	0.280
C20:5n3, EPA	0.08	0.07	0.08	0.02	0.757
C22:0	0.00	0.00	0.00	—	—
C22:1n9	0.18	0.19	0.20	0.03	0.856
C22:2	0.05	0.04	0.04	0.01	0.164
C23:0	0.05	0.05	0.04	0.003	0.377
C24:0	0.02	0.01	0.04	0.01	0.162
C22:6n3, DHA	0.13	0.1	0.12	0.04	0.783
C24:1n9	0.08	0.09	0.1	0.02	0.935
ω‐3 fatty acid	0.81	0.72	0.77	0.03	0.132
ω‐6 fatty acid	11.41^ab^	10.33^b^	11.61^a^	0.34	0.265
ω‐6: ω‐3	14.09	14.28	15.29	0.38	0.153
ΣSFA	40.03^b^	41.74^a^	39.60^b^	0.44	0.031
ΣUSFA	59.97^a^	58.26^b^	60.40^a^	0.44	0.030
ΣMUFA	47.28	46.78	47.55	0.25	0.266
ΣPUFA	12.69^ab^	11.48^b^	12.84^a^	0.37	0.278
MUFA/SFA	1.18^a^	1.12^b^	1.20^a^	0.02	0.065
PUFA/SFA	0.32^ab^	0.28^b^	0.32^a^	0.01	0.168
Unknown	0	0	0	—	—
Iodine Value	61.41^a^	59.07^b^	61.95^a^	0.66	0.104
Total FA (IMF)	100	100	100	—	—

*Note:* Fatty acid: C10:0 (Capric acid); C12:0 (Lauric acid); C14:0 (Myristic acid); C14:1 (Myristoleic acid); C15:0 (Pentadecylic acid); C16:0 (Palmitic acid); C17:0 (Margaricacid); C17:1 (Heptadecenoicacid); C18:0 (Stearic acid); C18:1isomer (Octadecenoicacidisomer); C18:1n‐9 (Oleic acid); C18:1cis‐11 (Vaccenic acid); C18:2n‐6 (Linoleic acid); C18:3n‐6 (γ‐linolenic acid); C18:3n‐3 (α‐linolenic acid); C20:0 (Arachidic acid); C20:1 (Gadoleic acid); C20:2n‐6 (Eicosadienoic acid); C20:3n‐6 (Dihomo‐γ‐linolenicacid); C20:4n‐6 (Arachidonic acid); C20:5n‐3 (Eicosapentaenoic acid); C24:0 (Lignoceric acid); C22:6n‐3 (Docosahexaenoic acid). Control (CON), basal diet; Stearic acid (SA); CON+0.50% SA; Palm oil (PO), CON+2% PO. SEM, Standard Mean Error. Within a row, values with different superscripts (a–b) differ significantly (*p* < 0.05) and trend (*p* < 0.10).

The effects of SA and PO on the fatty acid profile of pork belly lean tissue of growing‐finishing pigs is summarized in Table 4. The crude fat content and the most individual fatty acids, including short‐ and medium‐chain saturated fatty acids (e.g., C6:0 to C14:0), did not differ significantly across treatments (*p* > 0.05). However, pigs receiving the SA diet had significantly higher (*p* = 0.003) margaric acid (C17:0) compared to the CON (0.31%) and PO (0.32%) groups. Moreover, stearic acid (C18:0) content was also significantly affected by treatment (*p* = 0.047), being highest in the SA group (12.49%) and lowest in the PO group (10.07%). A significant treatment effect was observed for dihomo‐linolenic acid (C20:3n6) (*p* = 0.018), though absolute differences were small (0.04% across groups). Regarding total fatty acid classes, pigs fed SA had a significantly higher proportion of ΣSFA (39.49%) and a lower proportion of ΣUSFA (60.51%) compared to those fed PO (ΣSFA: 36.85%, ΣUSFA: 63.15%) (*p* = 0.019 and *p* = 0.018, respectively). Similarly, the PUFA/SFA ratio was significantly lower in the SA group (0.21) and higher in the PO group (0.25) (*p* = 0.006), indicating a shift toward greater saturation with the SA diet. The MUFA/SFA ratio was also significantly affected (*p* = 0.049), being highest in the PO group (1.47) and lowest in the SA group (1.32). However, the iodine value, which reflects the degree of unsaturation in fat, was significantly lower in the SA group (57.64) and higher in the PO group (60.60) (*p* = 0.004), further confirming the more saturated lipid profile associated with stearic acid. Nevertheless, no significant differences were observed in the levels of omega‐3 and omega‐6 fatty acids or in the ω‐6:ω‐3 ratio (*p* > 0.05), indicating that neither SA nor PO significantly influenced the PUFA balance in lean tissue.

**TABLE 4:** Effect of stearic acid vs. palm oil on fatty acid profile (pork belly, lean) in growing‐finishing pigs.

Items	CON	SA	PO	SEM^2^	*p*
Crude fat	46.50	47.19	46.53	1.26	0.898
C4:0	0.00	0.00	0.00	—	
C6:0	0.12	0.12	0.13	0.01	0.915
C8:0	0.02	0.03	0.03	0.01	0.700
C10:0	0.07	0.07	0.07	0.001	0.405
C11:0	0.00	0.00	0.00	—	—
C12:0	0.31	0.31	0.30	0.004	0.360
C13:0	0.00	0.00	0.00	—	—
C14:0	1.92	1.95	1.93	0.04	0.839
C14:1	0.04	0.04	0.04	0.01	0.953
C15:0	0.35	0.36	0.34	0.03	0.848
C15:1	0.00	0.00	0.00	—	—
C16:0	23.43	23.46	23.29	0.44	0.940
C16:1	2.66	2.67	2.66	0.01	0.656
C17:0	0.31^b^	0.35^a^	0.32^b^	0.01	0.003
C17:1	0.20	0.22	0.21	0.02	0.662
C18:0	11.31^ab^	12.49^a^	10.07^b^	0.57	0.047
C18:1, t	0.04	0.04	0.04	0.002	0.311
C18:1, c	48.68^ab^	47.59^b^	49.53^a^	0.49	0.160
C18:2n6t	0.04	0.04	0.04	0.004	0.634
C18:2n6c, LA	7.26	6.93	7.67	0.23	0.129
C18:3n6	0.03	0.03	0.03	0.003	0.274
C18:3n3, ALA	0.32	0.31	0.35	0.02	0.431
C20:0	0.19	0.18	0.20	0.01	0.652
C20:1	1.25	1.33	1.27	0.05	0.494
C20:2	0.29	0.30	0.29	0.03	0.982
C20:3n6	0.04^ab^	0.04^b^	0.04^a^	0.002	0.018
C21:0	0.00	0.00	0.00	—	—
C20:3n3	0.07	0.06	0.07	0.004	0.191
C20:4n6	0.03	0.03	0.03	0.001	0.405
C20:5n3, EPA	0.12	0.11	0.12	0.01	0.408
C22:0	0.07	0.07	0.05	0.01	0.609
C22:1n9	0.15	0.15	0.16	0.02	0.818
C22:2	0.05	0.04	0.04	0.01	0.364
C23:0	0.06	0.07	0.07	0.01	0.729
C24:0	0.04	0.05	0.05	0.01	0.771
C22:6n3, DHA	0.46	0.44	0.45	0.01	0.693
C24:1n9	0.08	0.14	0.11	0.03	0.494
ω‐3 fatty acid	0.97	0.92	0.99	0.02	0.270
ω‐6 fatty acid	7.41	7.07	7.82	0.24	0.128
ω‐6: ω‐3	7.63	7.66	7.91	0.25	0.712
ΣSFA	38.18^ab^	39.49^a^	36.85^b^	0.39	0.019
ΣUSFA	61.82^ab^	60.51^b^	63.15^a^	0.39	0.018
ΣMUFA	53.09^ab^	52.17^b^	54.02^a^	0.5	0.161
ΣPUFA	8.73	8.34	9.13	0.24	0.127
MUFA/SFA	1.39^ab^	1.32^b^	1.47^a^	0.03	0.049
PUFA/SFA	0.23^ab^	0.21^b^	0.25^a^	0.01	0.006
Unknown	0	0	0	—	—
Iodine Value	59.08^b^	57.64^c^	60.60^a^	0.33	0.004
Total FA (IMF)	100	100	100	—	—

*Note:* Fatty acid: C10:0 (Capric acid); C12:0 (Lauric acid); C14:0 (Myristic acid); C14:1 (Myristoleic acid); C15:0 (Pentadecylic acid); C16:0 (Palmitic acid); C17:0 (Margaricacid); C17:1 (Heptadecenoicacid); C18:0 (Stearic acid); C18:1isomer (Octadecenoicacidisomer); C18:1n‐9 (Oleic acid); C18:1cis‐11 (Vaccenic acid); C18:2n‐6 (Linoleic acid); C18:3n‐6 (γ‐linolenic acid); C18:3n‐3 (α‐linolenic acid); C20:0 (Arachidic acid); C20:1 (Gadoleic acid); C20:2n‐6 (Eicosadienoic acid); C20:3n‐6 (Dihomo‐γ‐linolenicacid); C20:4n‐6 (Arachidonic acid); C20:5n‐3 (Eicosapentaenoic acid); C24:0 (Lignoceric acid); C22:6n‐3 (Docosahexaenoic acid). Control (CON), basal diet; Stearic acid (SA); CON+0.50% SA; Palm oil (PO), CON+2% PO. SEM, Standard Mean Error. Within a row, values with different superscripts (a–b) differ significantly (*p* < 0.05) and trend (*p* < 0.10).

The effects of SA and PO on the fatty acid composition of pork loin lean tissue of growing‐finishing pigs are summarized in Table 5. Similar to pork belly fat, there were no notable differences observed in crude fat content in pork loin lean. Also, there were no significant differences observed in the individual short‐ and medium‐chain fatty acids (C4:0 to C14:0) and long‐chain saturated fatty acids such as palmitic acid (C16:0) and arachidic acid (C20:0) by dietary (*p* = 0.910 and *p* = 0.865, respectively) treatments. However, pigs fed the SA diet had a significantly higher proportion of stearic acid (C18:0) (13.50%) compared to both CON (11.06%) and PO (9.79%) groups (*p* = 0.001). Among MUFA, oleic acid (C18:1c) showed significantly higher levels in the PO group (47.26%) and the lowest in the SA group (44.07%) (*p* = 0.050). Total MUFA content followed a similar trend, being significantly higher in the PO group (51.81%) and lower in the SA group (48.56%) (*p* = 0.044). Consequently, the MUFA/SFA ratio was also significantly affected, being highest in the PO group (1.38) and lowest in the SA group (1.17) (*p* = 0.032). The PUFA such as linoleic acid (C18:2n6c), arachidonic acid (C20:4n6), and alpha‐linolenic acid (C18:3n3) were not significantly different among treatments (*p* > 0.05), but the PUFA/SFA ratio tended to be higher in the PO group (0.28) compared to the SA group (0.24), showing a trend toward significance (*p* = 0.085). Furthermore, ΣSFA were significantly increased in the SA group (41.67%) and reduced in the PO group (37.61%) (*p* = 0.034), while ΣUSFA followed the opposite trend (*p* = 0.033). This shift was reflected in the iodine value, which was significantly lower in the SA group (57.69) compared to the PO group (61.85) (*p* = 0.027), indicating a higher degree of saturation in pigs fed the SA diet. Also, there were no significant differences observed in the concentrations of total ω‐3 and ω‐6 fatty acids (*p* = 0.499 and *p* = 0.210, respectively), nor in the ω‐6:ω‐3 ratio (*p* = 0.326).

**TABLE 5:** Effect of stearic acid vs. palm oil on fatty acid profile (pork loin, lean) in growing‐finishing pigs.

Items	CON	SA	PO	SEM^2^	*p*
Crude fat	5.41	5.55	5.37	0.06	0.586
C4:0	0.00	0.00	0.00	—	—
C6:0	0.03	0.04	0.03	0.01	0.705
C8:0	0.02	0.02	0.01	0.01	0.721
C10:0	0.08	0.08	0.08	0.003	0.519
C11:0	0.00	0.00	0.00	—	—
C12:0	0.16	0.16	0.16	0.003	0.932
C13:0	0.00	0.00	0.00	—	—
C14:0	1.49	1.48	1.42	0.05	0.737
C14:1	0.02	0.02	0.01	0.01	0.542
C15:0	0.02	0.02	0.03	0.01	0.347
C15:1	0.00	0.00	0.00	—	—
C16:0	26.23	25.86	25.58	1.24	0.910
C16:1	3.70	3.59	3.62	0.16	0.906
C17:0	0.20	0.18	0.19	0.02	0.929
C17:1	0.16	0.15	0.17	0.01	0.465
C18:0	11.06^b^	13.50^a^	9.79^b^	0.46	0.001
C18:1, t	0.01	0.01	0.02	0.01	0.916
C18:1, c	45.40^ab^	44.07^b^	47.26^a^	0.82	0.050
C18:2n6t	0.09	0.09	0.08	0.01	0.827
C18:2n6c, LA	8.70	8.27	9.02	0.24	0.208
C18:3n6	0.00	0.00	0.00	—	—
C18:3n3, ALA	0.60	0.51	0.54	0.03	0.247
C20:0	0.15	0.16	0.15	0.004	0.865
C20:1	0.72	0.69	0.70	0.04	0.872
C20:2	0.32	0.30	0.29	0.03	0.770
C20:3n6	0.15	0.13	0.17	0.01	0.162
C21:0	0.00	0.00	0.00	—	—
C20:3n3	0.41	0.42	0.41	0.03	0.923
C20:4n6	0.05	0.04	0.05	0.01	0.453
C20:5n3, EPA	0.01	0.01	0.01	0.004	0.548
C22:0	0.00	0.00	0.00	—	—
C22:1n9	0.02	0.02	0.02	0.01	0.733
C22:2	0.00	0.00	0.00	—	—
C23:0	0.12	0.12	0.10	0.01	0.138
C24:0	0.06	0.06	0.06	0.01	0.653
C22:6n3, DHA	0.00	0.00	0.00	—	—
C24:1n9	0.00	0.00	0.00	—	—
ω‐3 fatty acid	1.01	0.95	0.97	0.04	0.499
ω‐6 fatty acid	8.99	8.53	9.32	0.25	0.210
ω‐6: ω‐3	8.90^b^	9.04^ab^	9.73^a^	0.21	0.326
ΣSFA	39.63^ab^	41.67^a^	37.61^b^	0.95	0.034
ΣUSFA	60.37^ab^	58.33^b^	62.39^a^	0.95	0.033
ΣMUFA	50.04^ab^	48.56^b^	51.81^a^	0.87	0.044
ΣPUFA	10.33	9.77	10.58	0.31	0.269
MUFA/SFA	1.26^ab^	1.17^b^	1.38^a^	0.05	0.032
PUFA/SFA	0.26^ab^	0.24^b^	0.28^a^	0.01	0.085
Unknown	0	0	0	—	—
Iodine Value	59.95^ab^	57.69^b^	61.85^a^	0.89	0.027
Total FA (IMF)	100	100	100	—	—

*Note:* Fatty acid: C10:0 (Capric acid); C12:0 (Lauric acid); C14:0 (Myristic acid); C14:1 (Myristoleic acid); C15:0 (Pentadecylic acid); C16:0 (Palmitic acid); C17:0 (Margaricacid); C17:1 (Heptadecenoicacid); C18:0 (Stearic acid); C18:1isomer (Octadecenoicacidisomer); C18:1n‐9 (Oleic acid); C18:1cis‐11 (Vaccenic acid); C18:2n‐6 (Linoleic acid); C18:3n‐6 (γ‐linolenic acid); C18:3n‐3 (α‐linolenic acid); C20:0 (Arachidic acid); C20:1 (Gadoleic acid); C20:2n‐6 (Eicosadienoic acid); C20:3n‐6 (Dihomo‐γ‐linolenicacid); C20:4n‐6 (Arachidonic acid); C20:5n‐3 (Eicosapentaenoic acid); C24:0 (Lignoceric acid); C22:6n‐3 (Docosahexaenoic acid). Control (CON), basal diet; Stearic acid (SA); CON+0.50% SA; Palm oil (PO), CON+2% PO. SEM, Standard Mean Error. Within a row, values with different superscripts (a–b) differ significantly (*p* < 0.05) and trend (*p* < 0.10).

## 4 Discussion

Fat supplementation in monogastric animals offers high energy density and FAs, contributing to efficient growth and productivity (Verge‐Mèrida et al. 2021). In the current study, SA and PO group pigs had no significant effects on growth performance, BFT, or meat quality traits in growing‐finishing pigs over a 12 week period and these findings are consistent with De la Llata et al. (2001); Apple et al. (2009); and Mazzoleni et al. (2023) who indicate that moderate dietary lipid manipulation often does not markedly influence growth parameters when energy intake is not compromised. In contrast to our research, Lee et al. (2013) found lower ADFI and higher feed efficiency in pigs receiving a PO supplemented diet. The absence of differences in growth performance implies that both SA and PO were well tolerated and provided sufficient dietary energy to support normal growth without any adverse effects. Backfat thickness and carcass weight were similarly unaffected by lipid diets, and this result aligns with Kouba et al. (2003) and Biswas and Kim (2025) who observed no significant alterations in carcass composition in pigs fed diets with different fat sources. This also correlates with Mitchaothai et al. (2007) and Hoque et al. (2025) who found no detrimental effects in either BFT or carcass traits. It can be speculated that under balanced dietary conditions, the type of dietary fat may have a minimal influence on adipose deposition and carcass yield. In terms of meat quality traits, no significant differences were observed among groups in WHC, cooking loss, or drip loss, which agrees with the findings of Alonso et al. (2012), Park et al. (2012), and Corino et al. (2002) who found limited effects on pork meat quality with dietary lipids. Although the PO group showed a numerically lower WHC and higher drip loss on day 7 postmortem, these values did not reach statistical significance. This trend may be associated with the fatty acid profile of PO, which contains higher saturated and certain MUSA that can influence muscle membrane stability and postmortem water retention (Wood et al. 2008). However, without statistical support, these observations remain speculative and warrant further investigation with a larger sample size or longer feeding period to detect subtle changes. The inclusion of dietary fats is a valuable strategy to enhance pork quality and optimize its FA composition to meet nutritional objectives (Leikus et al. 2018). The present study clearly demonstrates that inclusion of SA in pigs’ diet significantly alters the fatty acid composition of pork belly fat, belly lean, and loin lean by promoting a shift toward increased lipid saturation. This trend was characterized by elevated levels of stearic acid (C18:0), higher ΣSFA, and reduced MUFA and PUFA. These findings align with previous studies suggesting that long‐chain saturated fatty acids such as stearic acid are readily deposited in tissue lipids with limited metabolic modification, particularly in monogastric animals like pigs (Wood et al. 2008; Kouba and Mourot 2011). In pork belly fat, the SA diet led to significantly greater stearic acid content and a corresponding decrease in MUFA/SFA ratio and iodine value, indicating a more saturated fat profile. This can be attributed to the limited activity of Δ9‐desaturase (stearoyl‐CoA desaturase), the enzyme responsible for converting C18:0 to oleic acid (C18:1n9), in porcine adipose tissue (Bee, 2001). As a result, excess dietary stearic acid accumulates rather than being desaturated, thereby lowering the overall unsaturation of the fat. Although linoleic acid (C18:2n6c) tended to be higher in the PO group and lower in the SA group, differences were not statistically significant. Long‐chain PUFAs such as EPA and DHA remained unchanged, likely due to minimal dietary presence and limited endogenous synthesis in pigs (Wood et al. 2008). Similarly, pork belly lean tissue of the SA treated group showed increased margaric acid (C17:0) and stearic acid levels. We suppose that the increase in margaric acid is linked to altered microbial synthesis or differences in absorption dynamics. The SA group exhibited significantly higher ΣSFA and lower ΣUSFA, PUFA/SFA, and MUFA/SFA ratios, along with reduced iodine values, indicating enhanced saturation in intramuscular fat. These changes are not only relevant to the nutritional profile but also influence technological properties such as fat firmness, oxidative stability, and shelf life of pork products (D'Souza et al., 2005; Enser et al., 2000). While no significant differences were observed in ω‐3 or ω‐6 fatty acid levels, the minor significant increase in dihomo‐γ‐linolenic acid (C20:3n6) in the SA group suggests that dietary fat type may subtly affect fatty acid metabolism pathways. Yet, there was no study to compare the results; thus, further investigation is needful. The pork loin lean tissue also followed this trend. SA supplementation led to a significant increase in stearic acid and ΣSFA content, with a concurrent decrease in MUFA and PUFA levels. Notably, oleic acid (C18:1c), the predominant MUFA was significantly higher in the PO group and lowest in the SA group, confirming the influence of dietary lipid source on tissue fatty acid deposition. The PO‐fed pigs consistently showed higher MUFA/SFA and PUFA/SFA ratios, and elevated iodine values across tissues, indicating a more unsaturated and nutritionally favorable profile (Teye et al. 2006). These differences are likely attributable to the presence of palmitic, oleic, and linoleic acids in palm oil (Gunstone 2013; Warnants et al. 1998). Despite these compositional shifts, no significant changes were observed in total ω‐3 or ω‐6 fatty acids or in the ω‐6:ω‐3 ratio across treatments, suggesting that neither SA nor PO significantly affected the essential fatty acid balance under the conditions. Taken together, these results confirm that dietary fat source plays a pivotal role in determining the fatty acid composition of pork, with SA promoting a more saturated profile and PO contributing to greater unsaturation. While a saturated fat profile may enhance certain processing traits such as firmness and oxidative stability, it may also reduce the nutritional quality of pork fat from a human health perspective (Simopoulos, 2008). Conversely, higher unsaturation in the PO group may support more favorable health attributes but could increase susceptibility to oxidation. Therefore, the choice of dietary lipid source in swine diets should be strategically aligned with production goals, processing requirements, and target consumer preferences.

## 5 Conclusion

Our study demonstrates that dietary fat source plays a critical role in modulating the fatty acid composition of pork. Specifically, stearic acid supplementation in growing‐finishing pigs promotes a firmer and more saturated fat profile, which may benefit meat processing traits such as firmness, oxidative stability, and shelf life. However, this shift toward higher saturated fat content may be less favorable from a human health perspective due to associations with cardiovascular risk. Conversely, palm oil enhances the deposition of monounsaturated fats, particularly oleic acid, leading to a more unsaturated fat profile with improved nutritional attributes for consumers. These findings offer valuable guidance for tailoring swine diets to meet specific production goals, whether prioritizing meat processing efficiency or aligning with consumer demand for healthier meat products.

## Declaration of Generative A in Scientific Writing

During the preparation of this work, we used Chat‐GPT to improve readability and language. After using this tool/service, the author(s) reviewed and edited the content as needed and take full responsibility for the content of the publication.

## Author Contributions

**Vetriselvi Sampath:** data curation (lead), formal analysis (lead), investigation (lead), methodology (lead), resources (lead), writing – original draft (lead), writing – review and editing (lead). **Eunju Ko:** software (supporting), writing – original draft (supporting), writing – review and editing (supporting). **Jong Sang Yoo:** conceptualization (lead), writing – original draft (supporting), writing – review and editing (supporting). **Jemin Ahn:** data curation (supporting), formal analysis (supporting), methodology (supporting), writing – original draft (lead), writing – review and editing (supporting). **In Ho Kim:** conceptualization (equal), project administration (lead), supervision (lead), validation (lead), visualization (lead), writing – review and editing (supporting).

## Conflicts of Interest

The authors declare no conflicts of interest.

## Data Availability

The data that support the findings of this study are available from the corresponding author upon reasonable request.
